# Metabolic memory: a vascular perspective

**DOI:** 10.1186/1475-2840-9-51

**Published:** 2010-09-14

**Authors:** Thomas W Jax

**Affiliations:** 1 Profil Institut für Stoffwechselforschung, Hellersbergstrasse 9, 41460 Neuss, Germany; 2 Herzzentrum Wuppertal, Medizinische Klinik 3, Helios Klinikum, Universität Witten/Herdecke, Germany

## Abstract

Multiple and complex pathways promote the deleterious effects of hyperglycemia in diabetes, ultimately leading to micro- and macrovascular disease. Some of the known mechanisms in diabetic vascular disease may explain the initiation of the "metabolic memory", but fall short if long periods of time are involved.

Vascular research has been prolific in the past in finding links between microvascular dysfunction and subsequent macrovascular disease. Thus, this text will extend the current discussion of the "metabolic memory" by including available data from vascular research.

The hypothesis proposes that structural and functional changes in the microcirculation interact within the vascular continuum with larger arteries. This interaction may lead to subsequent *upstream *endothelial dysfunction, atherosclerosis and vascular complications ("Micro/Macro Interaction"). The underlying microvascular structural changes may be more long-term and possibly mediate the "metabolic memory".

This hypothesis, that the "not-so new" interaction between micro-and macrovasculature may promote "metabolic memory" effects extends and unifies currently discussed theories.

## The vascular perspective of the metabolic memory

In vascular research it is well established, that microvascular dysfunction eventually leads to subsequent macrovascular disease [1]. A dilation of prearterioles and arterioles results in an increase of shear stress, which triggers flow dependent dilation in conductance arteries [1,2], less shear stress secondary to microvascular dysfunction may lead to less vasodilation and thus endothelial dysfunction in conductance arteries.

In diabetes, hyperglycemia promotes both micro- and macrovascular damage by activating a network of multiple and complex pathways. Many of these may explain the initiation of the "metabolic memory" but fall short if long periods of time are involved. Structural changes in the microcirculation alter microvessels and their function, resulting in microvascular endpoints in the most susceptible organs.

The interaction within the vascular continuum between microvessels and larger arteries may lead to subsequent *upstream *endothelial dysfunction, atherosclerosis and vascular complications ("Micro/Macro Interaction"). In addition local intra vessel wall microvascular changes can promote localized atherosclerosis. These underlying microvascular structural changes may be more long-term and possibly mediate the "metabolic memory". The fact, that early intensive treatment of diabetes may be superior to intensification at a later disease stage indicates an early time window one has to make use of for intensive therapy in order to achieve a long-lasting therapeutic benefit by signifying the role of a structural fixation up to a "point of no return", when a regression of vascular disease progression is no longer possible. This extended, or rather unifying hypothesis will promote new research in this exiting area.

## Good metabolic control mediates long-term benefit

One of the major principles of the clinical long-term management of patients with diabetes mellitus is to prevent cardiovascular (CV) complications. Recently it was observed in several large scale clinical trials, that an intensive antihyperglycaemic treatment of diabetic patients, both in type 1 and 2, reduces the incidence of microvascular complications [3-5]. After the end of each study the participants were followed without any further intervention and, despite no further treatment differences, the advantage of intensive antidiabetic treatment persisted and even extended to a reduction of macrovascular events. The underlying cause of this phenomenon remains unclear; it is currently being discussed to be a "metabolic memory" effect: A defined period of good metabolic control conciliates a long term beneficial effect on cardiovascular endpoints. Several hypotheses, in particular the effects of a prolonged decreased oxidative challenge, are discussed. This comment extends the present view by introducing the role of macro- and microvascular interaction as a possible cause mediating "metabolic memory".

The global epidemic of diabetes leads to growing numbers of secondary cardiovascular complications. Cardiovascular mortality affects the majority of diabetic patients, the relative risk as compared to a non-diabetic population is several fold higher [6,7].

Tight metabolic control is the obvious approach to reduce the rate of cardiovascular events. Two very recent trials, ACCORD and ADVANCE, triggered a discussion, whether very tight metabolic control is really beneficial or not even contra productive [8,9]. Both trials explored in a large number of diabetic patients, whether an aggressive treatment algorithm to achieve good metabolic control can reduce micro- and/or macrovascular endpoints. The ADVANCE trial, although not finding a reduction of macrovascular endpoints, could show a reduction of diabetic nephropathy [8]. ACCORD was prematurely stopped because of higher mortality in the intensive treatment arm [9]. The underlying cause of these findings now is a matter of intense and controversial discussion. Both studies included diabetic patients with a history of cardiovascular disease, thus a more "advanced" subset of patients, in which the disease process might not be reversible.

Two other landmark studies showed differing results with regard to cardiovascular outcome [4,5]: although the initial results of the UKPDS study in newly diagnosed type 2 diabetic people [5] showed an improvement only in microvascular endpoints in the intensive treatment arm, the follow-up observation of another 10 years could also show a reduction of macrovascular endpoints and CV mortality in this group [4], despite of equal metabolic control (HbA1c) of both treatment arms after the end of the initial treatment period. The DCCT/EDIC trials showed very similar results for type 1 diabetic patients [3,10], including 1441 patients with a maximum of 5 years of type 1 diabetes and no complications.

The results of these studies led to a number of conclusions and new hypotheses, among which the concept of "metabolic memory" stands out. This hypothesis describes the observation that a prolonged tight metabolic control during early (newly diagnosed to 5 years) but not at later stage diabetes mellitus leads to sustained beneficial effects regarding micro- and macrovascular endpoints [3-5,10,11]. An earlier work by Garvey and co-workers [12] provided evidence for potential mechanisms. They demonstrated that an intensive insulin regimen in type 2 diabetic patients reversed the insulin resistance in muscle and liver as well as increased the beta cell insulin secretion without further use of insulin, claiming, that this would be caused by some "metabolic memory". In the present discussion the term "metabolic memory" implies that early glycaemic environment is "remembered" by target organs such as blood vessels, but also retina, heart, kidney, etc.[13]. The evolution of this concept has been recently discussed by Ceriello et al [13]. Briefly, the first reports of Kern reported two decades ago in a diabetic dog model, that poor glycaemic control followed by a period of good control did show the same rate of retinopathy as poorly controlled dogs [14]. Interestingly, Roy showed a shift of gene expression towards collagen IV and fibronectin expression in vitro (endothelial cells) and in diabetic rats (kidney cortex and myocardium) [15] with a similar design of a period with poor control followed by normalized glucose levels. These data implied that irreversible structural changes might be involved in the progression of late-term hyperglycaemic effects.

## The potential role of oxidative stress

Hyperglycemia induces a large number of alterations at the cellular level of vascular tissue that potentially accelerate the atherosclerotic process. Three major processes are thought to interrelate and promote vascular changes, which include glycolysation of proteins, oxidative stress and protein kinase C activation [16]. There exists a wealth of literature that highlights the role of oxidative stress in the development of vascular damage and subsequent atherosclerosis. Details are not within the scope of this paper and can be found elsewhere [17].

The potential relation between oxidative stress and the "metabolic memory" is presently the most discussed hypothesis [13]. This concept implies that a sustained exposure to reactive nitrogen and oxygen species (RNOS), mediates multiple downstream biological processes via covalent modification of proteins, nucleic acids and/or lipoproteins, which finally accounts for irreversible deterioration. On the other hand, a transient RNOS production on the short-term is an integral part of cellular signal transduction under physiological conditions [13].

Although this hypothesis is compelling, it would not explain an effect, that according to EDIC and UKPDS "metabolic memory" extends to beyond 10 years. To explain macrovascular disease these mechanisms would have to occur within the vessel walls of the macrovasculature, especially within the endothelium as the central cellular compartment of early atherosclerotic development. One could argue that early RNOS overload primes for the later development of macrovascular disease, since atherosclerosis is generally not reversible.

Several recent papers explore the possibility of epigenetic changes induced by hyperglycemia.. El-Osta and co-workers were able to show that transient hyperglycemia induced long-lasting activating epigenetic changes in aortic endothelial cells in vitro and in vivo in non-diabetic mice [18,19]. Various methylation or dymatylation events may lead to upregulation of NFκB-p65, which may lead to subsequent activation of pro-inflammatory pathways, possibly leading to micro- and macrovascular disease [20]. Tonna et al propose the involvement of epigenetic changes especially for the development of diabetic nephropathy [20]. Although this approach is very compelling, more in vivo data will be needed. As vascular endothelium is an organ with a high turnover and regenerative capacity [21,22], the question remains unanswered how early endothelial changes mediated through oxidative stress and/or epigenetic changes can account for long-term macrovascular risk. Additionally, multiple mechanisms including induction of an antioxidative response and counter regulation of gene expression exist to maintain endothelial integrity [23-26].

Reality is probably more complex and thus the current hypothesis of the "metabolic memory" may be extended by (re-)introducing a vascular perspective. A central observation of EDIC and UKPDS is, that diabetic people who were recruited early in their disease career (newly diagnosed to 5 years), despite of treatment modalities, first presented with microvascular disease only whereas macrovascular disease occurred much later. Patients with intensive treatment developed less micro- and later also less macrovascular disease. These simplified observations may lead to the conclusion that there is a timely order, and that microvascular disease may be a prerequisite for macrovascular complications in these patients.

## Micro- and Macrovasculature

The above findings are supported by observations that microcirculatory changes correlate with macrovascular atherosclerosis and the occurrence of cardiovascular events. Wong and co-workers found in the "Atherosclerosis Risk in Communities"-study (ARIC), that retinal arteriolar narrowing as an established marker of microvascular damage is closely associated with the risk of events related to coronary heart disease [27]. Although these effects were predominantly seen in women, they were independent of hypertension and diabetes mellitus. Other studies conformed this association between retinal changes and risk for coronary artery disease in general [28], micro-and macrovascular changes in diabetes [28,29], subclinical atherosclerotic disease [28] and the risk of stroke [28,30-32].

It is important to realize, that microvascular dysfunction is not limited to one organ but rather a systemic process [33,34]. In hypertension for example histopathological changes in retinal and coronary arteries are very similar [35,34,33]. There also exists a microangiopathic process within the vessel wall of conduit arteries resembling that of retinal changes [36], which are discussed to precede macrovascular changes. Thus microvascular changes in a particular vascular bed (e.g. retinal arterioles) may allow comparisons to other vascular regions.

## Fibrosis of the microvascular interstitium

**Figure 1:**
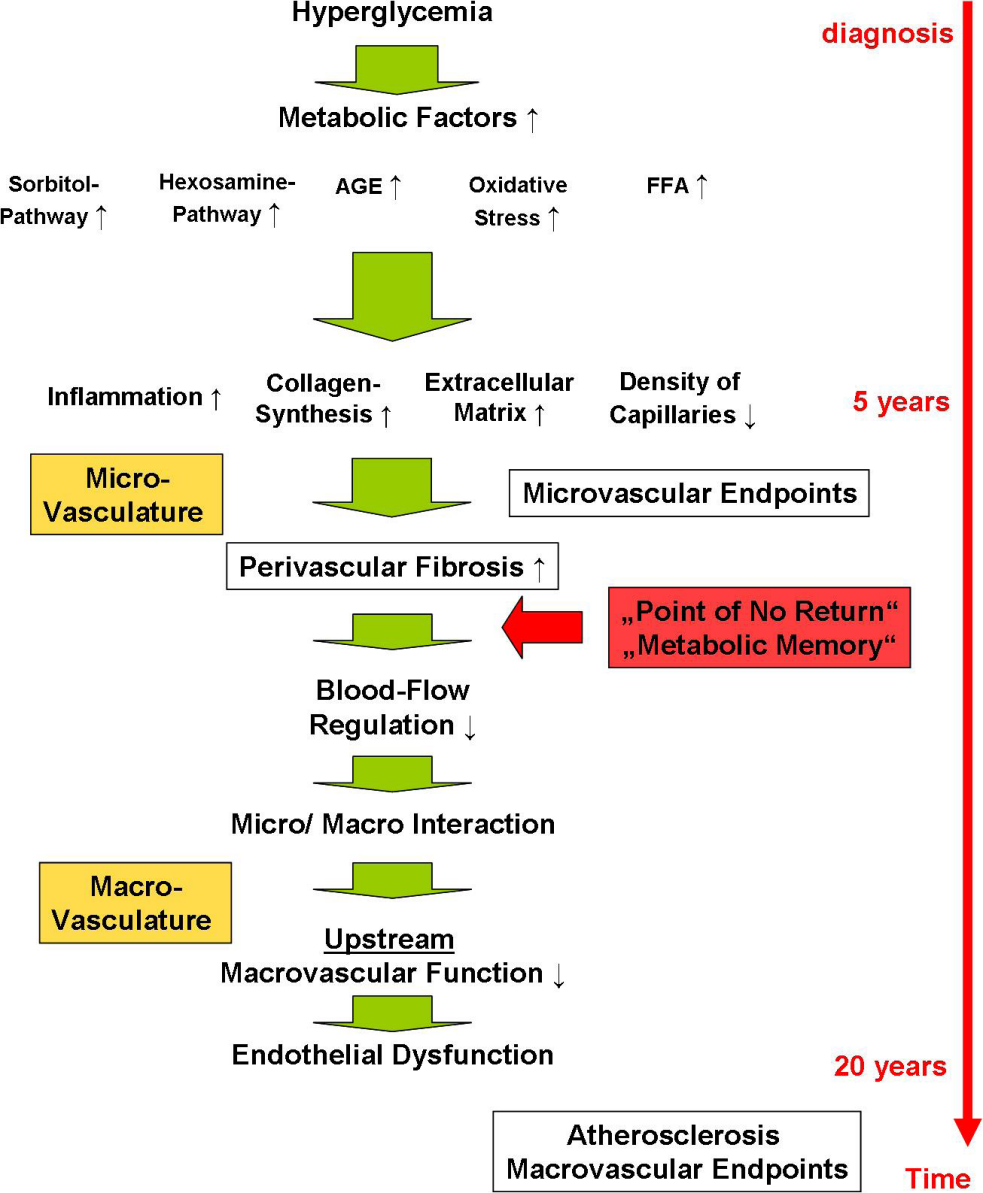
**Hyperglycemia and vascular outcome**. Hyperglycemia activates multiple and complex pathogenetically relevant pathways. Inflammatory processes lead to a thickening of the basal membrane of microvessels and perivascular fibrosis resulting in microvascular endpoints in the most susceptible organs, such as renal insufficiency and retinopathy. The structural changes also result in functional impairment and a reduction in blood flow regulation, which in turn reduces macrovascular endothelial function ("Micro/Macro Interaction"). The loss of endothelial protection may lead to the subsequent development of atherosclerosis. The structural changes within the microcirculation may account for building up a "metabolic memory".

Besides of functional changes of the microcirculation structural changes are mediating long term complications. As mentioned above, it is well established that hyperglycaemia mediates vascular changes through several pathways [37]. These pathways are interconnected with each other and include the increased synthesis of sorbitol and hexosamines, glycosylation of proteins, synthesis of "advanced glycosylation end products" (AGE), and oxidative stress (figure 1). The downstream effects in the microvasculature are mainly characterized by changes of the extracellular matrix composition and structure. Induction of inflammatory processes through receptor binding of AGEs or oxidative stress mediate changes of the structure of the extracellular matrix [37]. Subsequent fibrosis and the extension of extracellular structures impair capillary blood flow and in particular reduces capillary density (figure 1) [38-40]. Reduced blood flow may then facilitate microvascular complications. These become apparent the earliest in the most vulnerable organs, which are, among others, retina and kidney. Early diabetic microvascular changes relate to a thickening of the basement membrane in the capillaries. These changes occur throughout the whole body and include arterioles in the glomeruli, retina, skin, muscle, and myocardium, resulting in classic diabetic microangiopathy [41]. Various mechanisms have been proposed for diabetic microvascular complications [41], but the endothelium plays a major role in the disease processes. Surprisingly, endothelial cells show a high heterogeneity in structure and function throughout the vascular tree [42-44]. This relates to a variety of functional differences between different vascular beds, such as permeability, hemostasis, vasomotor tone, leukocyte transmigration and others. These may, at least in part, explain the susceptibility of certain organs to diabetic microvascular damage.

## The vascular continuum: Interaction between micro- and macrovasculature

**Figure 2:**
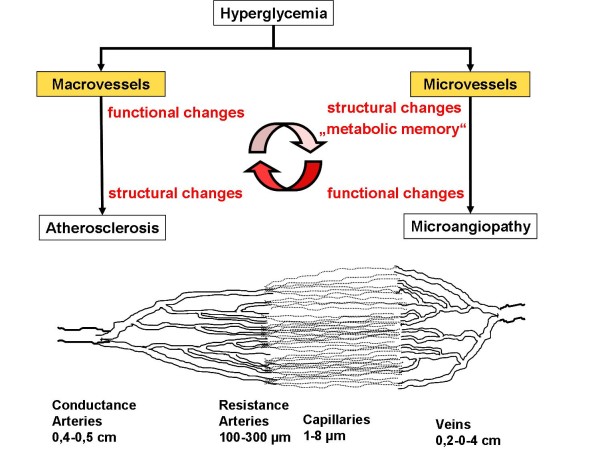
**The Vascular Continuum**. Conductance arteries mediate downstream effects to resistance arteries and capillaries. If microcirculation is impaired by perivascular fibrosis as in diabetes, upstream effects influence conductance arteries.

Conductance arteries provide sufficient blood flow to end organs, but blood demand is not regulated through dilatation of the larger arteries but rather through the alteration of microvascular resistance [45]. This function is located within the specific organ at the level of the resistance arteries or arterioles (figure 2). The microcirculation regulates vasomotion and permeability and can thus adapt blood flow according to local metabolic needs [46,47].

For example, coronary vascular resistance, which is well studied, under physiological conditions is mainly regulated through microvessels; conductance vessels only participate with about 5% to total coronary resistance [45,48-50]. A common misperception may be that there is only little or no interaction between macro- and microcirculation and *vice versa*, but rather the opposite is the case. Macrovasculature can influence downstream resistance arteries through several pathways: A hemodynamically relevant stenosis > 50% of lumen reduction can be compensated through lowering downstream vascular resistance metabolically (e.g. for maintenance of oxygen supply) [51,52]. This reduces the amplitude of the flow reserve, so that if needed, flow reserve may not be sufficient for adequate supply of the end organ, which may in turn lead to symptoms such as angina pectoris in the heart [53,54]. Also, repeated microembolisms may alter downstream vascular resistance (reviewed in [45]).

On the other hand microvasculature may also influence the function of upstream larger arteries. Patients with hypertensive heart disease present with a substantially reduced coronary flow reserve [55]. Coronary microangiopathy is associated with a reduction of upstream dilatatory function of epicardial coronary arteries [56]. This association is correlated to the degree of myocardial hypertrophy. The interface may, again, be endothelial function. Vasodilatory capacity of large arteries is mainly dependent on acute stimulation of nitric oxide synthesis of endothelial cells. Shear stress is one of the major regulators of local NO-synthesis. In principle, an increase of a normal microcirculatory flow-reserve will increase *upstream *flow mediated vasodilation by a factor 3-6 [45,57]. Microvascular dysfunction with a reduced flow reserve will lead to a reduced shear stress-induced NO synthesis in upstream macrovessels [58]. This implies that structural changes such as perivascular fibrosis of microvessels can directly influence upstream arteries and cause endothelial dysfunction (over time) and subsequent atherosclerosis (figures 1 and 2). This proposed mechanism may represent the cause of the "metabolic memory". Since the effect was only seen in diabetic patients who were included in studies shortly after their diagnosis, microvascular structural changes might represent a "point of no return", after which an intensive treatment might less effective or not effective at all. (figure 1).

Indirect studies suggest that a long-term reduction of microcirculatory function would lead to endothelial dysfunction and consequent atherosclerosis. Kelm and coworkers studied 62 hypertensive patients with myocardial microangiopathy and without any macrovascular coronary artery disease (CAD) for 10 years [45]. 28 patients developed relevant coronary artery disease, which would be the equivalent of an incidence for CAD of 8%. This is multiple times higher than the yearly incidence of CAD in American (0.37%) or Australian (0.11%) populations [45]. The risk of major cardiac events was 2- 3 fold higher.

In a recent review Oresanu and Plutzky hypothesize, that microangiopathy in the neovasculature of vaso vasorum may propagate and accelerate diabetic atherosclerosis [41]. The vaso vasorum is a network of microvessels in the adventitia and media of large arteries [59] providing the vascular wall of these large arteries with nutrients but also influence lipid deposition and metabolism and neurohumoral factors [59]. Hypoxia and macrophages within atherosclerotic plaques may promote intraplaque and intra vessel wall angiogenesis and alter vessel wall microcirculation [36]. This hypothesis would supplement the theory of this paper, in particular the occurrence of plaques at certain, rather specific locations such as bifurcations.

In addition, several authors suggested, that microvascular structure I not only the site of vascular resistance but probably also the origin of most of the wave reflections generating increased central blood pressure and thus influencing large artery stiffness [60]. Small artery remodelling can be observed in hypertensive diabetics as well as in patients with diabetes mellitus alone, underlining the role of diabetes for the pathophysiology of microvascular disease [61-63]. Drugs that somehow improve microvascular structure are also effective for improving the mechanical properties of large arteries [62]

## Conclusion

Although diabetes specific data is not yet available, there exist strong analogies to hypertensive vascular disease, which will allow well designed experiments and trials to test this hypothesis. It could be possible that microvascular events in the earlier course of the disease process may be reversible through adaption processes whereas macrovascular changes may be more irreversible through maladaptation.

Proof-of-concept studies as well as clinical studies will be needed to support this unifying concept, but a better understanding of the interplay of metabolism, micro- and macrovasculature may ultimately promote new therapeutic options and better patient outcome.

## Competing interests

The author declares that they have no competing interests.
